# Assessment of the deformation of the outer nuclear layer in the Epiretinal membrane using spectral-domain optical coherence tomography

**DOI:** 10.1186/s12886-019-1124-z

**Published:** 2019-05-17

**Authors:** Seiji Takagi, Shigeki Kudo, Hideo Yokota, Masahiro Akiba, Michiko Mandai, Yasuhiko Hirami, Masayo Takahashi, Yasuo Kurimoto, Masahiro Ishida

**Affiliations:** 1 Department of Ophthalmology, Teikyo University, University Hospital Mizonokuchi, 5-1-1 Futago, Takatsu-ku, Kawasaki, Kanagawa 213-8507 Japan; 2 Department of Ophthalmology, Kobe City Eye Hospital, 2-1-8, Minatojima-Minamimachi, Chuo-ku, Kobe, 650-0047 Japan; 3 Cloud-Based Eye Disease Diagnosis Joint Research Team, RIKEN Center for Advanced Photonics, 2-1, Hirosawa, Wako-shi, Saitama, 351-0198 Japan; 4 Image Processing Research Team, RIKEN Center for Advanced Photonics, 2-1, Hirosawa, Wako-shi, Saitama, 351-0198 Japan; 5 R&D Division, Topcon Corporation, Tokyo, Japan; 6 Laboratory for Retinal Regeneration, RIKEN Center for Biosystems Dynamics Research, 2-2-3 Minatojima-Minamimachi, Chuo-ku, Kobe, 650-0047 Japan

**Keywords:** Epi retinal membrane, Outer nuclear layer, Metamorphopsia, M chart

## Abstract

**Background:**

We aimed to investigate the deformation of the outer nuclear layer using optical coherence tomography in patients with epiretinal membrane (ERM) and its relationship with metamorphopsia.

**Methods:**

Thirty-nine eyes from 39 patients with ERM were included in the study. Patients with the subtypes of pseudo macula hole and lamellar hole were excluded. Twenty-one fellow eyes without macular disease were included as normal controls. Forty-nine B-scan images were obtained in the range of 20 degrees around the macula using SD-OCT. The outer nuclear layer (ONL) was evaluated as a three-dimensional image (3D-ONL) reconstructed using the distance between the ONL and retinal pigment epithelium (RPE) line. The deformation of the ONL was figured at the reference plane and evaluation plane (ONL-B). The characteristic parameters of the ONL-B were defined as circularity, area ratio, and axis ratio. The correlations between these parameters and visual acuity and MCHART score ratio (MH/MV) were then evaluated.

**Results:**

ONL height was significantly higher in ERM patients than in normal controls (54.1 ± 5.3 μm and 84.1 ± 12.9 μm, respectively; *P* < 0.001). In ERM patients, the MV score was 0.53 ± 0.50, the MH score was 0.71 ± 0.61, and the distance from the RPE line to the ONL-B was 153.5 ± 13.5 μm. The axis of the ONL-B in normal controls and ERM patients was − 6.25 ± 21.8 and − 1.28 ± 29.1, respectively, which indicates that the ONL is horizontally long in both normal individuals and ERM patients. The circularity and area ratio were significantly smaller in ERM patients than in normal controls. In all ERM patients, MH/MV had a significant correlation with axis (r = − 0.29, *p* = 0.034), circularity (r = − 0.28, *p* = 0.044), and area ratio (r = − 0.47, *p* = 0.001). Moreover, we found that the correlation was more significant if the subjects had an axis of the ONL within ±10 degrees (*n* = 16); the correlations of MH/MV with axis (r = − 0.29, *p* = 0.034), circularity (r = − 0.53, *p* = 0.021), and area ratio were more significant (r = − 0.78, *P* < 0.0001).

**Conclusion:**

The ONL is horizontally long in normal individuals and ERM patients. The direction of metamorphopsia is correlated with the direction of ONL deformation.

## Background

Idiopathic epiretinal membrane (ERM) is one of the most common vitreoretinal diseases. Contraction of the fibro-cellular membrane at the surface of the sensory retina and tangential traction of the retina lead to deterioration of visual acuity and cause metamorphosia [1–3].

Metamorphopsia is the most common symptom in patients with ERM [3], which causes moderate-to-severe distortion of vision that impacts patients’ daily life activities, such as walking difficulties and social distress. A study that used the 25-item National Eye Institute Visual Function Questionnaire has shown that the severity of metamorphopsia is strongly influenced by vision-related quality of life [4].

Removal of the ERM is a well-established surgical procedure [5], and additional removal of the internal limiting membrane has been reported to decrease the chance of recurrence of ERM years after surgery [6, 7]. In addition, significant visual acuity recovery after surgery has been documented in many reports [6, 8]. However, the longitudinal improvement in metamorphosis is not as noteworthy as that of visual acuity [9].

High-resolution spectral domain optical coherence tomography (SD-OCT), which is now the most common retinal imaging device, is widely used to investigate the changes of microstructure in the retina in patients with ERM.

The central retinal thickness [10], integrity of the ellipsoid zone (EZ) [11], and disruption of cone outer segment tips (COST) [12, 13] have been reported to be critical prognostic factors of visual acuity.

With regard to metamorphopsia, central foveal thickness, inner nuclear layer thickness, and outer nuclear layer (ONL) + outer OPL thickness have been reported to be correlated with the findings of the MCHART [9], which is the examination to quantify metamorphopsia in the horizontal and vertical lines through the macula.

The aim of the present study was to investigate the force exerted on the photoreceptor layer around the macula using OCT images. To this end, we observed the deformation characteristics of the ONL, which is closer to the photoreceptor in the retina.

## Methods

This retrospective observational study included consecutive patients with ERM who had undergone surgeries, and these patients were enrolled from March 2016 to January 2018 in Teikyo University School of Medicine, University Hospital Mizonokuchi, Kanagawa, Japan. An ERM was defined as a thin membrane attached to the surface of the retina, as detected in the SD-OCT images preoperatively. Eyes with long axial length (axial length > 27 mm), glaucoma, retinal vascular diseases, uveitis, or trauma and eyes that had undergone retinal surgery or photocoagulation previously were excluded. In addition, we excluded patients with the two subtypes of ERM, namely, pseudo macular hole and lamellar hole type. A fellow eye without the macular disease was regarded as the normal control.

### Image analysis

**Fig. 1:**
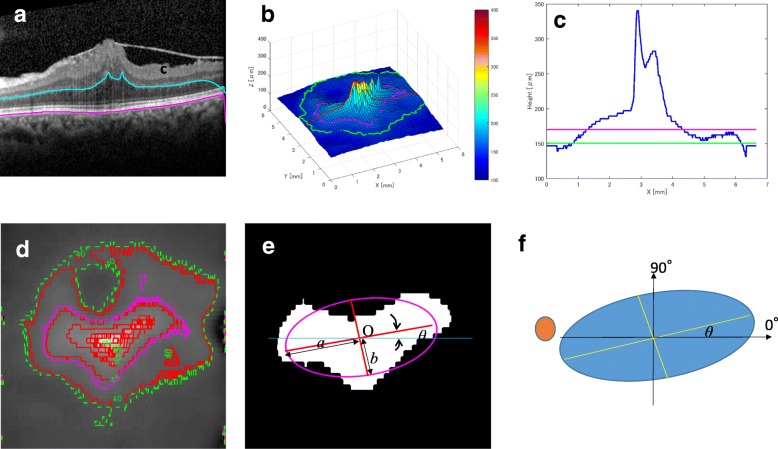
**a** Volumetric optical coherence tomography (OCT) data of the fovea from 49 slice B-scan images. The retinal pigment epithelium line and the boundary between the outer nuclear layer (ONL) and the outer plexiform layer (OPL) was outlined using the IOWA Reference Algorithm, with manual correction at areas of steep protrusion. **b** An example of a three-dimensional outer nuclear layer. The height indicates the distance from the RPE line. **c** The outer nuclear layer (ONL) sectional height profile on the horizontal line passing through the macula. The green line indicates the reference plane, and the purple line indicates the position of the ONL base (ONL-B). The percentage shows the ratio to the maximum thickness. **d** Two-dimensional image with the height as the intensity contour map. The green boundary is the reference plane and the purple boundary is the outer nuclear layer base (ONL-B). The actual height can be obtained by multiplying the result with 3.87 μm. **e** The outer nuclear layer base (ONL-B) and its approximate ellipse. The major axis length (a), minor axis length (b), center (O) of the ONL_B, and angle (θ) from the horizontal axis are calculated by the moment method, and an ellipse (purple) was fitted to the ONL_B. **f** The demographic how the angle was calculated on the approximate ellipse on the outer nuclear layer base (ONL-B). The brown circle describes that the disc is the cup on the fundus of the left eye. The angle θ is defined as a positive angle for counterclockwise

Retinal images were obtained using SD-OCT (Spectralis, Heidelberg Engineering, Heidelberg, Germany). The implementation of image analysis was conducted using MATLAB (2016b version; The MathWorks Inc., Natick, MA, USA). Forty-nine B-scan images with 512 slices with linear interpolation were obtained in the range of 20 degrees corresponding to the 6.7 × 6.7 × 1.5-mm region (pixel width x height x depth = 512 × 512 × 400 pixels) around the macula (Fig. 1a). Each B scan image was the average of 16 single B scans.

The boundary between the ONL and outer plexiform layer (blue line in Fig. 1a) and retinal pigment epithelium (RPE) line (purple line in Fig. 1a) was detected for volumetric OCT data using the IOWA Reference Algorithm. In the area of steep protrusions of the ONL, each segmentation line was outlined manually (Fig. 1a). The manual segmentation was checked by two investigators (S.T., S.K.) separately and was used for analysis. The ONL three-dimensional data (3D-ONL) were then re-constructed using the thickness between the ONL and RPE on each A scan. (Fig. 1b) This image was composed of a width and height of 512 pixels and defined as “HI”..

The reference plane was defined as the section at the level of maximum frequency of a 3D-ONL histogram, which is indicated by the green lines in Fig. 1b–d. However, the boundary of the reference plane was very rough, which made analysis of its morphological features difficult; this is because there were fewer vertical data (49 slices in 6.7 mm) than horizontal data (512 scan in 6.7 mm), and because there were many convexo-concavity parts due to the complex contractile force.

Therefore, the region of interest, i.e., the ONL-Base (ONL-B) was set as follows:

1. The boundary of the ONL_B exists within the HI.
2. Area A of the concave portion of the ONL_B is 20,000 or less by pixel unit.
3. The A/A0 ratio is 1.2 or less. The denominator (A0) is area of the ONL_B.
4. The A1/A0 ratio is 0.95 or more; the numerator (A1) is an area restored from the elliptic Fourier method [14] by changing the number of points on the boundary of the ONL_B, from 100, 50 to 25 with five fixed frequency terms.
5. The area of the ONL_B and the perimeter of ONL-B was calculated as S, L.
6. Next, from the moment method, the major axis length (a), minor axis length (b), and center of the ONL_B were calculated, and then the elliptic curve of the ONL-B was generated (Fig. 1e).
7. ***Axis*** (θ in Fig. 1e and f) was defined as the angle from the horizontal axis of the macula, and the leftward orientation was set to “+.”

To remove size and orientation effects, we focused on the following characteristic parameters related to the deformation of 3D-ONL: ***circularity*,** ***area ratio,*** and ***axis ratio***.

***Circularity*** was calculated as *4 π S / L* ^*2*^ using the elliptic Fourier method after normalization. This value indicates that closer to circle is closer to 1.0.

***Area ratio*** was calculated as *S/(ab)*. The area ratio of the ONL-B was figured as an approximated ellipse. This value indicates how the contour line of the ONL-B is irregular.

***Axis ratio*** was calculated as *a/b*; this parameter indicates whether the shape was slender or not.

### Visual function

Best corrected visual acuity was obtained using the Landolt C charts, and then these values were then converted to the logarithm of the minimum angle of resolution (logMAR) equivalent for statistical comparisons.

MCHART (Inami Co., Tokyo, Japan) is a subjective test to quantify metamorphopsia on the horizontal and vertical dot lines. The gaps between the dots are in the range of a viewing angle of 0.2° to 2°, and the subjects obtained the minimum viewing angle to recognize the dot shift at a distance of 30 cm horizontally (horizontal MCHART score [MH]) and vertically (vertical MCHART score [MV]) [3]. The ratio of MH and MV (MH/MV) was then calculated (If the MH or MV score was 0, it was recorded as 0.05).

### Statistical analysis

The mean scores were compared and SD values were calculated for each parameter of visual function and OCT measurements. A parametric test was performed to compare the patient characteristics and each OCT parameter between ERM patients and normal controls. The associations between the MCHART scores, BCVA, and OCT parameters were examined by using Pearson correlation, t-test, and hypothesis testing for null hypothesis. A *P* value of 0.05 was considered statistically significant. Statistical analyses were performed using statistical software (SPSS®, version 21.0; SPSS Science, Chicago, Illinois, USA).

## Results

### Patients

In total, 68 eyes from 68 patients were enrolled, and those with eyes with long axial length (*n* = 8), lamellar hole (*n* = 7), pseudo macular hole (*n* = 12), and poor quality OCT images (*n* = 2) were excluded. Thirty-nine eyes from 39 patients with ERM and 21 eyes from 21 controls were included in the study.

The demographic data and OCT parameters in ERM patients and normal controls are shown in Table 1.

**Table 1:** Demographic data of ERM patients and normal controls

	ERM patients	Controls	*p* value
N	39	21	
Age (years)	68.1 ± 9.3	66.5 ± 11	
Sex	Male: 16	Male: 12	
VA	0.24 ± 0.16	−0.12 ± 0.03	*P* < 0.001
M chart			
MV	0.53 ± 0.50		
MH	0.71 ± 0.61		
ONL height (μm)	84.1 ± 12.9	54.1 ± 5.3	< 0.001
ONL-B			
Axis (degrees)	−1.28 ± 29.1	−6.25 ± 21.8	0.497
Circularity	0.79 ± 0.09	0.85 ± 0.07	0.006
Area ratio	0.74 ± 0.03	0.75 ± 0.02	0.010
Axis ratio	1.54 ± 0.37	1.51 ± 0.35	0.798

*VA* visual acuity, *MV* vertical MCHART score, *MH* horizontal MCHART score, *ONL* outer nuclear layer

No significant differences were noted in the age and sex ratios between the two groups.

The preoperative BVCA in ERM patients and normal controls was 0.24 ± 0.16 and − 0.12 ± 0.03, respectively (P < 0.001).

In ERM patients, the MV was 0.53 ± 0.50 (range, 0–2.0), and the MH was 0.71 ± 0.61 (range, 0–2.0).

The height of the ONL in ERM patients was significantly higher than that of normal controls (54.1 ± 5.3 μm and 84.1 ± 12.9 μm, respectively; *P* < 0.001). The distance from the RPE line to the ONL-B was 153 ± 13.5 μm.

**Fig. 2:**
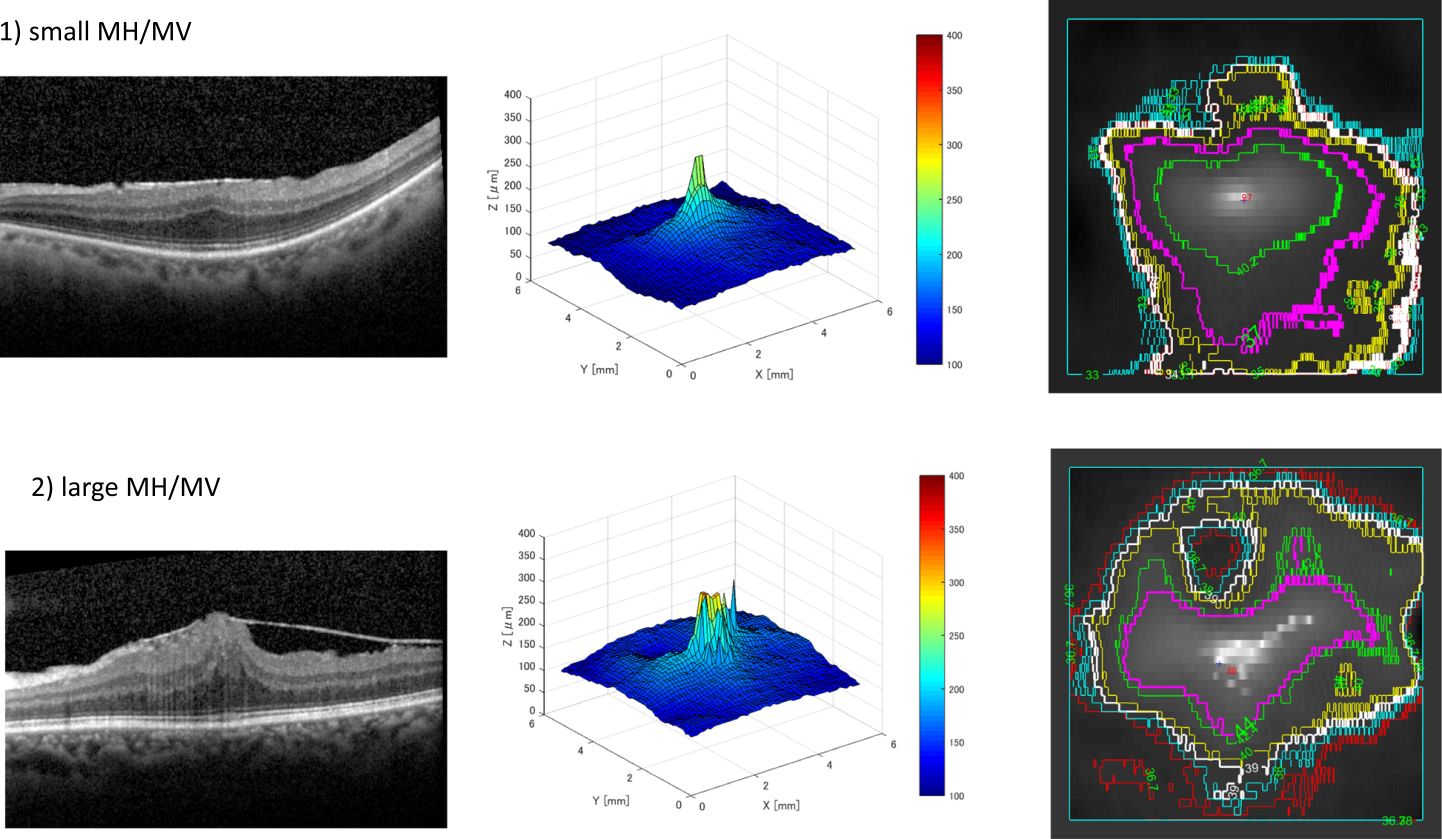
Representative cases of small MH/MV and large MH/MV. The upper row shows a representative case of a left eye with a small MH (0)/MV(0.7). The best corrected visual acuity (BCVA) is 0.16, circularity is 0.79, and axis ratio is 1.15, which indicate that the boundary of the ONL_B was more circular. The lower row shows a representative case of a left eye with a large MH(1.3)/MV(0). The best corrected visual acuity (BCVA) is 0.40, circularity is 0.66, and axis ratio is small (1.15). The boundary of the ONL_B was a narrower elliptic shape than the case of the upper row. The circularity is 0.65, area ratio is 0.66, and axis ratio is approximately 2, which indicate that the boundary of the ONL_B was longer horizontally

**Fig. 3:**
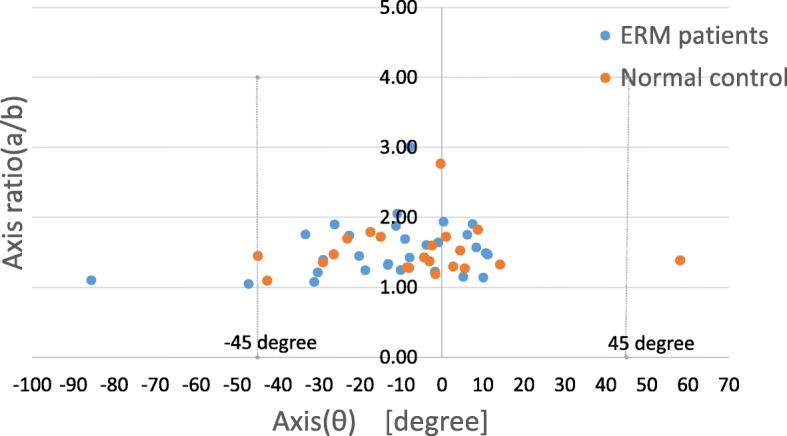
Relationship between the axis and axis ratio. Most of the data are in the range of − 45 degrees to 45 degrees, and the average θ value is − 3.02 ± 26.7 degrees

The relationship between the axis and the axis ratio is shown in Fig. 3. The axis of the ONL-B in normal controls was − 6.25 ± 21.8 and the axis ratio was 1.51 ± 0.35, which indicate that the ONL is naturally horizontally long. In addition, the axis of the ONL-B in ERM patients was − 1.28 ± 29.1. Thirty-five of 39 eyes had an axis of ONL-B within ±45 degrees and the axis ratio was 1.54 ± 0.37, which indicate that the ONL were likely to deform to be horizontally long (Fig. 2).

The characteristic parameters of the ERM patients and normal controls were as follows: circularity: 0.79 ± 0.09 and 0.85 ± 0.07, respectively, *p* = 0.006; area ratio: 0.74 ± 0.03 and 0.75 ± 0.02, respectively, *p* = 0.01; axis ratio: 1.54 ± 0.37 and 1.51 ± 0.35, respectively, *p* = 0.79).

The correlation between the ONL parameters and MH/MV is shown in Table 2.

**Table 2:** Correlation between the ONL parameters and MH/MV

	Correlation with MH/MV			
	All ERM (*n* = 39)		Long axis within ±10 degrees (*n* = 15)	
	r	*p* value	r	*p* value
ONL height (μm)	0.02	0.883	0.11	0.702
ONL-B.				
Axis	−0.04	0.796	− 0.39	0.147
Circularity	−0.28	0.044	−0.53	0.021
Area ratio	−0.47	0.001	−0.78	0.000
Axis ratio	0.31	0.028	0.32	0.123

*MV* vertical MCHART score, *MH* horizontal MCHART score, *ONL* outer nuclear layer

ONL height did not correlate with MH/MV. In all ERM patients (*n* = 39), MH/MV had a significant correlation with axis (r = −0.29, *p* = 0.034), circularity (r = − 0.28 *p* = 0.044), and area ratio (r = − 0.47, *p* = 0.001). Moreover, the long axis was limited within ±10 degrees from the horizontal axis (*n* = 15), and the correlation of MH/MV with the ONL parameters circularity (r = − 0.53, *p* = 0.021) and area ratio (r = − 0.78, *p* = 0.000) were more significant.

## Discussion

The present study showed that the ONL layer around the macular area is naturally horizontally long, and that it is more prominent in ERM patients than in normal controls.

The photoreceptor layer, the first set of neurons involved in light perception, consists of the retinal pigment epithelium (RPE), outer segment,(OS), inner segment (IS), cell bodies, and long axons. SD-OCT enables the visualization of these structures in vivo via the white and black intensity images. A recent immunobiological study has shown that the hypo-reflective wide layer over the ELM line is considered to be the cell body and the long axons of the photoreceptor [15]. In that study, the ONL-B was around 150 μm from the RPE line, which indicated that the ONL-B was evaluated at the level of the cell body and the Henle nerve fiber layer of the photoreceptor [15].

The present study showed that the ONL-B is naturally horizontally long, and it is more evident in ERM patients (Fig. 3). This horizontal long structure is consistent with the form of the nerve fiber layer, the temporal fibers are dispersed widely, and a small percentage of arcuate fibers are dispersed horizontally [16]. In the present study, the ONL-B is more evident in ERM patients, the average axis was 2 degrees from the horizontal line, and 90% of ERM patients showed an axis within ±45 degrees, which may be due to the ERM contraction direction. Our colleague recently reported that the vertical retinal displacement was greater than the horizontal retinal displacement, as assessed by measuring the position of the retinal vessels in ERM patients [17], which indicates that retinal morphology is likely to deform to be horizontally long with ERM vertical contraction. The results of the present study are consistent with this observation.

Classically, the origin of metamorphopsia was considered to be the displacement of the cones, which in turn, causes a false localization of the image [18]. Macropsia and micropsia, special subtypes of metamorphopsia, show that the retinal displacement is correctly logically associated with the symptom, outward retinal displacement-derived macropsia [19], and inward retinal displacement-derived micropsia [20]. Moreover, Amsler suggested that outer retinal and choroidal changes cause metamorphopsia and scotoma [1]. We assumed that the original reason of metamorphopsia was displacement of the photoreceptor by ERM contraction, and the direction of ONL (a much bigger volume) deformation would correlate with the direction of the force on the photoreceptor.

Many clinical studies using retinal images have investigated the cause of metamorphopsia [21], and several previous studies support that changes of the photoreceptor layer are responsible. Okamoto et al. reported that preoperative MCHART score was significantly correlated with the ONL + OPL thickness using SD-OCT images [9]. Ooto et al. reported that cone morphologic feature patterns in the foveal photoreceptor layer was associated with metamorphopsia, as assessed using adaptive optics scanning laser ophthalmoscopy images [22].

aIn this study, circularity is the parameter indicating how the ONL is close to a perfect circle, and area ratio is the parameter indicating whether the contour line of the ONL-B is irregular. MH/MV is negatively correlated with circularity, which indicates that an increase in MH influences the deformation of the ONL in the vertical direction. Area ratio is higher in ERM patients, which indicates that the ONL in ERM patients has a more complicated deformation with contraction.

We found that the correlation between the metamorphopsia parameters and morphological characteristic parameters is more significant in cases where the long axis is close to vertical (which is ±10°). This result may indicate that metamorphopsia contains different direction components of distortion, and that the MCHART would be affected by the component orthogonal to their axis. Arimura et al., who developed the MCHART test, reported a significant correlation between horizontal contraction of the retina and the MV score and between vertical contraction of the retina and the MH score by measuring the superficial retinal position [23], and our result is consistent with their report.

We observed the characteristics of the outer retina and their effect on metamorphopsia. However, several recent reports documented that the inner nuclear layer was significantly correlated with metamorphopsia [9]. [19] Moreover, we observed metamorphopsia in patients with diseases of the superficial retinal layer, such as small branch vein occlusion and superficial small ERM. Therefore, we think that the mechanism of metamorphopsia involves multiple factors, including the conscious effect [24], and the pathology of metamorphopsia is different in each disease.

ERM could be classified on the basis of macular morphology into retinal thickening, pseudo hole, and lamellar hole [25]. In the subtype of retinal thickening, which is the most frequently observed type of ERM, membrane-attached macular and tangential traction destroyed the macular morphology. In contrast, in fovea-sparing ERM types, such as the pseudo hole type, the membrane does not cover the foveal area and has minimal influence on macular morphology; it has also been reported to show well-preserved foveal function. We included only patients with the retinal thickening type in the present study to focus on the influence of deformation direction on metamorphopsia direction.

There are limitations in this study. This was a retrospective study on a limited number of participants. The IOWA Reference Algorithm does not work well automatically for a region where the ONL steeply rises. Therefore, we had to perform manual segmentation for these parts. New algorithms may be needed to enable segmentation of OCT images in diseased eyes.

## Conclusions

In conclusion, in this study, we showed that the ONL in the macular area is naturally horizontally long. The deformation characteristics were significantly correlated with metamorphopsia, as revealed by the MCHART score. MH is affected more by vertical contraction than by horizontal contraction by the ERM.

## Abbreviations

3D-ONL: ONL three-dimensional data
AMD: Age-related macular degeneration
ERM: Epiretinal membrane
EZ: Ellipsoid zone
IS: Inner segment
MH: Horizontal MCHART score
MV: Vertical MCHART score
ONL: Outer nuclear layer
ONL-B: ONL-Base
OS: Outer segment
RPE: Retinal pigment epithelium
SD-OCT: Spectral domain optical coherence tomography
